# Cell Death in Escherichia coli: Incomplete Base Excision Repair under Depletion of DapB and Dxr Proteins

**DOI:** 10.1128/mbio.01611-22

**Published:** 2022-06-29

**Authors:** Reha Onur Azizoglu

**Affiliations:** a Department of Gene and Cell Therapy and Food Engineering, Mediterranean (Akdeniz) University, Antalya, Turkey

**Keywords:** cell death, *Escherichia coli*, incomplete base excision repair, oxidative damage, stress response

## Abstract

The generation of reactive oxygen species (ROS) within the cell is a significantly shared aspect of bacterial cell death against different stress conditions. The main cell death mechanism due to the generation of reactive oxygen species is then the incomplete base excision repair (BER) in response to oxidized nucleotides. In their recent article in *mBio*, C. C. Gruber, V. M. P. Babu, K. Livingston, H. Joisher, and G. C. Walker (*mBio* 13[1]:e03756-21, 2022) report two new stress conditions regarding the depletion of DapB and Dxr, which indeed cause similar mechanisms for cell death. These two stress conditions trigger highly distinctive stress response mechanisms within the cell, but the ultimate cell death mechanism is a result of a shared process. These findings prove that the disturbance in the homeostasis of cells under a variety of different stresses initiates cell death mechanisms through the production of ROS, generation of 8-oxo-dG and the incomplete BER.

## COMMENTARY

The emergence of antibiotic-resistant bacteria consistently poses a significant threat to global public health. One key aspect to overcome this global threat is to develop effective antimicrobial strategies. Although the direct effect of antimicrobial agents on the target organisms and their mode of action are well characterized, the specific bacterial response mechanisms against these antimicrobial agents and how they lead to cell death has not yet been well defined. Therefore, it is crucial to enhance our knowledge of the mechanisms involved in bacterial cell death to increase the efficacy of newly developed antibiotics.

Exposure to bactericidal antimicrobials is reported to trigger enhanced production of highly toxic hydroxyl radicals within the cell that leads to its death regardless of bacteria being Gram-positive or Gram-negative (1). The mechanism of cell death through the generation of endogenous ROS is poorly understood, and it used to be regarded as a direct consequence of an increase in ROS concentrations. In contrast, the main ROS (superoxide (O_2_-) along with the hydrogen peroxide combination (H_2_O_2_)) can react with cellular constituents at a limited level (2). Therefore, it cannot be the only mechanism that leads to cell death. It is suggested that the level, as well as the target of Fenton oxidants (reactive hydroxyl radicals and intermediates generated when H_2_O_2_ goes through Fenton reaction), mainly lead to the oxidation of nucleotides and nucleic acids (2). Then, these Fenton oxidants particularly damage the nucleotides, and the guanine is promptly oxidized to 8-oxo-dGTP due to this exposure. Incorporation of these oxidized nucleotides into the genome leads to mutations because of their undesired pairing ability with dA and dC. In the following phase, bacteria either block this defect by degrading the 8-oxo-dGTP avoiding its incorporation into the genome or by repairing the defect in the genome through the action of base excision repair (BER) glycosylases of MutM and MutY (3). Moreover, the BER process has a significant potential to cause DNA lesions, and thereby the cell becomes prone to cell death through double-strand breaks in the DNA (4). On the other hand, incomplete BER could take part in cellular death under a variety of different stress conditions depending on the environmental and bacterial status. Although cell death through nucleotide oxidation and BER is observed in response to various modes of antibiotics, its specific role in cell death against different stress conditions is still poorly understood (Takahashi et al. 2017).

In this issue, Gruber et al. (3) specifically shed light on how and when an incomplete BER pathway takes part in bacterial cell death under newly described stress conditions regarding the depletion of DapB and Dxr proteins. For this purpose, the authors screen a large number of proteins that are essential for the aerobic growth of bacteria from an Escherichia coli essential protein degradation library. In this method, the single essential protein is basically depleted through the action of an inducible protease. Further screening of the library reveals two novel proteins that are not previously reported to protect cells against death through the incomplete BER pathway. The first protein of the two, DapB (4-hydroxy-tetrahydrodipicolinate reductase), is a protein that is involved in the lysine biosynthetic pathway (5). The second identified protein, 1-deoxy-d-xylulose 5-phosphate reductoisomerase, Dxr, is involved in the isoprenoid biosynthesis pathway (2). Therefore, both of these proteins are involved in pathways that are targeted in antibiotic development efforts. From this perspective, diaminopimelate (DAP) is an essential metabolite for bacteria since it is involved in lysine biosynthesis and cross-linking of peptidoglycan. In E. coli, DAP biosynthesis involves successive enzymatic reactions. This process involves the reactive production that is carried out by DapB protein, which is an immediate precursor of DAP (6). Since DapB is essential for cell wall synthesis, its depletion shows similar stress response patterns to certain antimicrobials (such as β-lactams), which also targets cell wall synthesis.

In the case of Dxr depletion, the observed response within this particular study is quite different from the previously reported stress factors. The authors reveal that the depletion of Dxr results in the defect of cells for not being able to produce new quinones as a result of the blockage that is caused by the isoprenoid biosynthesis. When the Dxr is depleted, cells express genes that are involved in anaerobic respiration systems and show the acidification of their cytoplasm.

The depletion of both of these proteins leads to an increase in 8-oxo-dG by indicating an increase in the oxidative stress response of the bacteria. In parallel, an increase in 8-oxo-dG is also a marker for incomplete BER-mediated cell death (4, 6, 7). The authors report that the depletion of DapB and Dxr within a single depletion strain can cause differential expressions of repair processes due to incorrect stoichiometry. This indicates that BER is a tightly regulated process, and the intermediates formed during the repair process can create toxic effects on the cell. For instance, MutY complements and repairs the action of MutM by removing the mismatched and undamaged adenine residues. But, the depletion of DapB causes a drastic increase in its expression, and the depletion of Dxr leads to a slight decrease. The disproportional changes in the expression of genes of BER most likely result in an incomplete BER, which leads to cell death. Interestingly, Gruber et al. (3) highlight that the overexpression of 8-oxo-dGTP sanitizer of MutT has a protective effect on the cells when either DapB or Dxr is depleted. These observations indicate the fact that the primary cause of cell death under the depletion of DapB and Dxr is the incorporation of increased 8-oxo-dG in the genome through the use of 8-oxo-dGTP.

To further confirm the role of incomplete BER in cell death under these conditions, the authors construct a double mutation in two BER-associated genes, *mutM* and *mutY*. Excitingly, this double mutation decreases the degree of cell death when either DapB or Dxr is depleted. These findings confirm that oxidative stress and incomplete BER play a significant role under the stress conditions that are caused by the depletion of these two proteins.

The transcriptomic comparison of DapB and Dxr depleted cells mostly reveal distinct transcription levels with a few similarities. In one sense, the depletion of DapB strongly activates the Rcs regulon, which is also involved in response to β-lactam antibiotics. While the depletion of Dxr slightly activates the Rcs regulon, the components of the acid stress systems are strongly upregulated. Interestingly, the transcription of responsible genes of iron uptake is affected by the depletion of both DapB and Dxr. Iron is an essential component of Fenton reactions and the generation of Fenton radicals. The DapB depletion leads to an increased expression of *entB* that is responsible for an increased uptake of iron and increased cellular iron levels proportionally. On the other hand, the Dxr depletion process results in the downregulation of several uptake systems and upregulation of the ferrous iron transport system (3).

Another significant difference in bacterial responses against depletion of these two proteins includes lower expression of *soxS* under DapB depletion and increased expression of it under Dxr depletion. SoxS is reported to act as an activator for superoxide dismutase stress response genes by eliminating the superoxide from the cell after being involved in the oxidative stress response of bacteria (8). Both the incomplete BER and the damage caused by oxidative stress can be repaired up to a certain extent through the *recA* knockout mutant that is constructed by the authors. The findings indicate that the RecA-mediated repair pathway is initially effective against the damaging effects of depletion; however, it loses its effect once the damage becomes overwhelming.

Broadly speaking; this study excitingly shows two distinct stress conditions causing different physiological changes in cells lead to cell death through a shared incomplete BER mechanism. It is noteworthy to say that the degree of cell death through an incomplete BER pathway significantly varies between these two characterized stress factors. This proves the fact that both the level and the route of the stress factor on cells’ physiological state determines the level of ROS production. Moreover, the degree of disturbance on the cells’ machinery affects the 8-oxo-dG-related DNA repair. These findings prove that the ultimate cell death is a result of incomplete BER rather than the direct effect of an increase in the concentration of ROS within the cell. Thus, two leading yet previously unidentified stress conditions of cell death are the key findings of this study. The cellular function of these two proteins, DapB and Dxr, differs from each other and the physiological response of the cell against depletion of them varied tremendously. This highlights the fact that ROS production is not a single pathway solution within the cell, and it is a shared metabolic outcome of stressed cell physiology under a variety of different stresses. This is an essential proof of disturbance of the homeostasis of a cell due to exposure to a variety of different stress conditions (including exposure to antibiotics) that initiate cell death mechanisms through the production of ROS, generation of 8-oxo-dG, and the incomplete BER. The similarity in incomplete BER mediated cell death pathway between distinct stress conditions can be detrimental to the development of effective antibiotic treatments in the era of increased development of antibiotic resistance among pathogenic bacteria and treatment of bacterial infections.
